# RNAi to treat SARS‐CoV‐2—variant proofing the next generation of therapies

**DOI:** 10.15252/emmm.202215811

**Published:** 2022-03-14

**Authors:** Nigel A J McMillan, Kevin V Morris, Adi Idris

**Affiliations:** ^1^ Centre for Cell and Gene Medicine Menzies Health Institute Queensland Griffith University Southport QLD Australia

**Keywords:** Microbiology, Virology & Host Pathogen Interaction, Pharmacology & Drug Discovery

## Abstract

There is an urgent need to bring new antivirals to SARS‐CoV‐2 to the market. Indeed, in the last 3 months, we have seen at least two new antivirals approved, molnupiravir and paxlovid. Both are older established antivirals that show some efficacy against SARS‐CoV‐2. The work by Chang *et al* (2022) in the current issue of *EMBO Molecular Medicine* explores the use of short interfering RNAs to directly target SARS‐CoV‐2 and shows that RNAi is an effective approach to reducing, or even eliminating viral replication, depending on the experimental setting. This antiviral effect results in significant prevention of infection‐related pathology in animals. The key feature of this approach, besides its simplicity as naked siRNAs, is that all current variants are covered by this treatment.

## Graphical abstract

N. McMillan, K. Morris, and A. Idris discuss development of RNAi‐based therapy against SARS‐CoV‐2 infection as reported by PC. Yang and colleagues, in this issue of *EMBO Mol Med*.

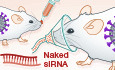

This exciting work (Chang *et al*, 2022) builds on a number of papers showing *in vitro* effectiveness of siRNAs to target and inhibit SARS‐CoV‐2 (Tolksdorf *et al*, 2021; Ambike *et al*, 2022) and two previous publications with *in vivo* treatments (Fig 1) in Syrian hamsters (Khaitov *et al*, 2021) and K18‐hACE2 mice (Idris *et al*, 2021).

**Figure 1:**
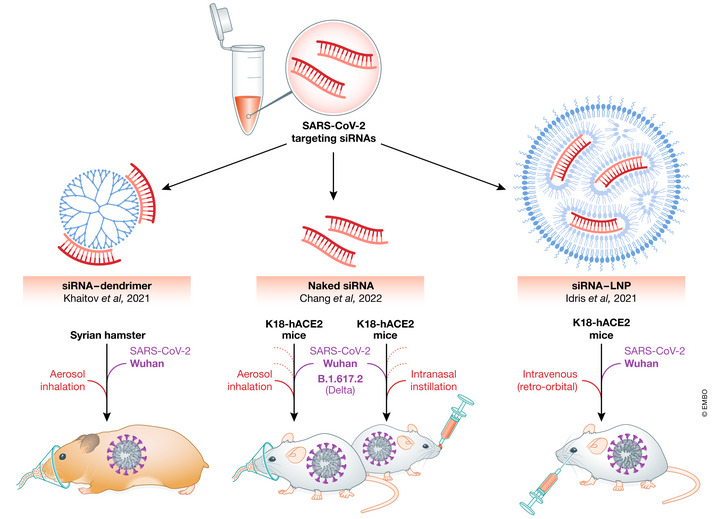
Small RNA repression of SARS‐CoV‐2 *in vivo* Various approaches employed and found to repress SARS‐COV‐2 *in vivo* and repress COVID‐19 disease are shown schematically.

In the hamster model, siRNA was given via inhalation using a dendrimer complex as carrier and was found to result in a 54% reduction in virus titres after 6 days, whereas others found using the mouse model that intravenous injection of siRNA packaged lipid nanoparticles repressed virus in lungs and inhibited COVID‐19 disease. The key difference with the current work, however, is that they used intranasally administered naked siRNAs, a method much more amenable to outpatient or in‐home treatment, and achieved complete repression if treatment began before infection, or a 96% reduction in virus load if treatment was started at the same time as infection. They also show good activity against current variants Alpha, Delta, Gamma, and Epsilon.

Why RNAi? Currently, there are no antivirals that directly target the SARS‐CoV‐2 RNA genome on the market. RNAi as an antiviral has long been explored with the original *in vivo* work of Sailen Barak’s team over 17 years ago showing potent inhibition of intranasally delivered siRNAs to treat RSV and parainfluenza virus (Bitko *et al*, 2005). Indeed, a number of clinical trials have taken place since then. The most well‐known was Alynlam’s ALN‐RSV01, an RSV siRNA that showed potent effects in humans but was ultimately abandoned after missing the primary endpoint in a phase 2b study (Gottlieb *et al*, 2016). There are many advantages to exploring RNAi as an antiviral, chiefly among them is speed. Drug development takes time and virus can mutate to avoid traditional protein inhibitors making it incredibly challenging to develop a drug for a variant that may not have arisen yet. RNAi is a “plug and play technology” that can be adapted and quickly amended.

The current crop of SARS‐CoV‐2 antivirals have been developed from research programs targeting a range of other viruses and repurposed for today’s pandemic demand. These are first generation drugs taken to market quickly due to the urgent unmet need and for which we would expect to see much better versions in future. Remdesivir was developed by Gilead and partners to treat RNA viruses and function by targeting the unique RNA‐dependent, RNA polymerase. It was active against SARS‐COV‐1 and MERS and found to also work against SARS‐CoV‐2 (Wang *et al*, 2020). Molnupiravir is a nucleoside analog that causes copying errors by the virus and was originally developed at Emory University to treat influenza while paxlovid is a combination of nirmatrelvir and ritonavir that inhibits the viral protease and was originally targeted to HIV. Mutation is the enemy of all antiviral drugs, and we are acutely aware of this viruses’ ability to mutate rapidly. RNAi is also susceptible to mutational escape, but the current siRNAs have been chosen to specifically target deeply conserved regions among not only current variants, but also the entire betacoronavirus family, which greatly reduces this risk.

Another "RNA" direct targeting approach being explored is CRISPR, which has recently been shown to target SARS‐CoV‐2 (Blanchard *et al*, 2021; Fareh *et al*, 2021). However pre‐existing antibodies to CRISPRs and the need to translate the packaged CRISPR mRNA and gRNA in virus infected cells may hinder the clinical translation of this approach. RNAi does not require translation of mRNA. It is programmable, scalable, and stable.

The outcome of all these studies is that RNAi is highly effective in treating SARS‐CoV‐2 *in vitro* and more importantly *in vivo*. However, the paper does raise an issue that all antivirals to SARS‐CoV‐2 must address and that is the timeliness of treatment. The sooner the treatment begins the better the virus repression and patient outcome. Experience with drugs like remdesivir shows that if treatment is too late, then these drugs are not clinically useful, and SARS‐CoV‐2 is particularly challenging as significant virus replication occurs before symptoms are apparent. However, besides treating early‐stage patients, antivirals will be beneficial in the prophylactic setting. For example, treating residents in an aged care setting where an outbreak is current, keeping frontline medical staff healthy, or for departing traveler's is more than enough reason to develop better antivirals against a virus that will be with us well into the future.
